# Convergence and divergence between the transcriptional responses to Zika virus infection and prenatal irradiation

**DOI:** 10.1038/cddis.2017.109

**Published:** 2017-03-16

**Authors:** Roel Quintens

**Affiliations:** 1 Radiobiology Unit, Institute of Environment, Health and Safety, Belgian Nuclear Research Centre, Mol, Belgium

In a recent paper in *Cell Death and Disease*, Di Cunto and co-workers found that infection of human neural progenitors (hNPCs) with Zika virus (ZIKV) induced transcriptional responses that were partly comparable to three different genetic mutations (*CitK*^−/−^, *Magoh*^+/−^, and conditional *Elp3*^−/−^ mice), which result in microcephaly in mice.^1, 2, 3, 4^ The highest overlap between these different conditions was found in the activation of P53 target genes.^4^

Previously, we identified a robust signature of 84 genes that are induced (differentially expressed and/or alternatively spliced) in the embryonic mouse brain and immature primary mouse cortical neurons after exposure to ionizing radiation.^5^ Also, in our study, we observed a very high overlap between our gene signature, comprising mostly predicted (both established and novel) p53 targets, and genes that were induced in the *Magoh*^+/−^ mouse model, with no less than 26 out of 55 induced genes from the *Magoh*^+/−^ model belonging to our 84-gene signature. This is considerably more than the 16 upregulated genes (out of 3434) found to overlap between *Magoh*^+/−^ mice and ZIKV-infected hNPCs.^4^ The induced genes in *Magoh*^+/−^ mice furthermore included several other genes, which were not retained in our robust signature, but were induced by ionizing radiation in either E11 mouse brains (*Ak1*), or primary mouse cortical neurons (*Fam38a*, *Gria3*, and *Psrc1*). Of note, this list of overlapping genes also included *Ano3* and *Arap2*, which were not considered to be p53 targets according to Di Cunto and co-workers.^4^ However, we have clearly shown that *Ano3* is a transcriptional p53 target both *in vivo* and *in vitro*,^5^ while *Arap2* expression was found to be dependent on the transcriptional activity of p53 in mouse embryonic fibroblasts.^6^ Because our experimental model of mice irradiated at embryonic day 11 (E11) with a dose of 1 Gy also results in severe microcephaly as a consequence of massive apoptosis,^7, 8^ we believe it is therefore also very relevant in the view of ZIKV-induced microcephaly.

With regard to the study of Di Cunto and co-workers, I have found that 30 genes that were upregulated in ZIKV-infected hNPCs also belong to our 84-gene signature (Figure 1a). This is a much more significant overlap (*P*=5.7E-5, hypergeometric overlap with 18 500 common protein coding genes between human and mouse as the total number of genes) than those with the two published studies in which microcephaly results from neuronal apoptosis (21/199 (*P*=0.99) in *CitK*^−/−^; 17/55 (*P*=8.5E-3) in *Magoh*^+/−^). Of these, 16 genes are uniquely in common between our signature and that of ZIKV-induced genes, and are enriched in functions related to aging (5 genes), cellular differentiation (8 genes), dendrite development (4 genes), and neuronal apoptosis (4 genes) (Figure 1b). In contrast, the remaining 14 genes that are also shared with the other microcephaly models are clearly related to p53-mediated apoptotis and DNA damage signaling (Figure 1c).

Moreover, and maybe even more interesting, 10 genes from our radiation-induced signature are downregulated in the ZIKV-infected hNPCs (Figure 1d). Four of these genes (*BTG2*, *C11orf182/DDIAS* and *GTSE1*) are known to be involved in cell cycle arrest (Figure 1e) or chromosome stability during mitosis (*CKAP2*, ref. 9) and are downregulated (*DDIAS*, *GTSE1*) or transiently upregulated (*BTG2*, *CKAP2*) during embryonic brain development (Figure 1f). In contrast, the remaining six genes (*C14orf159*, *C9orf116*, *IGDCC4*, *PQLC3*, *TM7SF3,* and *TMEM19*) have currently not been functionally characterized, but they do have in common that they are strongly and constitutively upregulated in the mouse embryonic brain between E9 and E16 (Figure 1f). This suggests that these genes might be primarily important for brain development or neuronal differentiation, rather than classical p53-regulated pathways, which are in general downregulated during brain development.^10^

Thus, our gene signature of radiation-responsive genes in the embryonic mouse brain and immature primary cortical neurons displays a very significant overlap with that of hNPCs that had been infected with ZIKV. Genes that are shared with other microcephaly models are most likely responsible for the observed p53-dependent apoptotic response, which is a common feature of the different models. The 16 genes that are exclusively upregulated in ZIKV-infected cells and irradiated brains, might then be involved in other p53-mediated processes such as, for instance, neuronal differentiation. However, this might also hold true for some of the downregulated genes, of which the function is currently unknown. In this respect, it is interesting to note that ZIKV infection has been implicated both with a delay of neurogenesis^11^ as well as with the secretion of cytokines at levels that can induce premature neuronal differentiation, leading to neuronal apoptosis and microcephaly.^12^

On the basis of their extensive upregulation during mouse brain development and neuronal differentiation, we hypothesized that the activation of some of these p53 target genes after irradiation might result in premature neuronal differentiation of neural progenitors.^5^ This hypothesis was contradicted by a more recent study of the *Magoh*^+/−^ mice, in which it was convincingly shown that apoptosis of neuronal progenitors was p53-dependent, whereas premature differentiation was not.^13^ Therefore, I have reanalyzed the transcriptional profiles of *Magoh*^+/−^ mice and prenatally irradiated mice (unpublished data). In this case, I investigated the gene expression profiles during embryonic mouse brain development (between E9 and E16) of overlapping genes, radiation-specific genes, and *Magoh*-specific genes. This analysis showed that both overlapping and radiation-specific genes were very significantly enriched during brain development. This was in sharp contrast with the *Magoh*-specific genes, which are almost all significantly downregulated at E16. Moreover, both overlapping and radiation-specific gene sets were very significantly enriched in p53 targets, while this was much less the case for the *Magoh*-specific genes. Finally, while overlapping genes were mostly related to p53-mediated DNA damage signaling, both radiation-specific genes as well as *Magoh*-specific genes did not show a clear functional enrichment. Thus, *Magoh* haploinsufficiency results in activation of two sets of genes: those that are developmentally upregulated, p53-mediated and related to apoptosis, and another gene set, which is developmentally downregulated. Radiation exposure apparently only activates p53 targets, of which the large majority are developmentally upregulated. This is an important difference between both mouse models, suggesting to us that in our model p53 may well be involved in (premature) neuronal differentiation by activating a specific subset of genes. Since many of these genes are also induced by ZIKV-infection, such as *BLOC1S2*, which has recently been shown to promote early neuron progenitor differentiation,^14^ they might have a similar outcome in that setting as well.

Altogether, this shows that prenatal exposure to ionizing radiation shares both convergent and divergent features with ZIKV infection, all of which may help to better understand the molecular mechanisms that underly their neuropathogenic outcome. We are currently investigating the function of some of these genes in order to better understand their possible implications in the etiology of congenital microcephaly, and brain development in general.

## Figures and Tables

**Figure 1:**
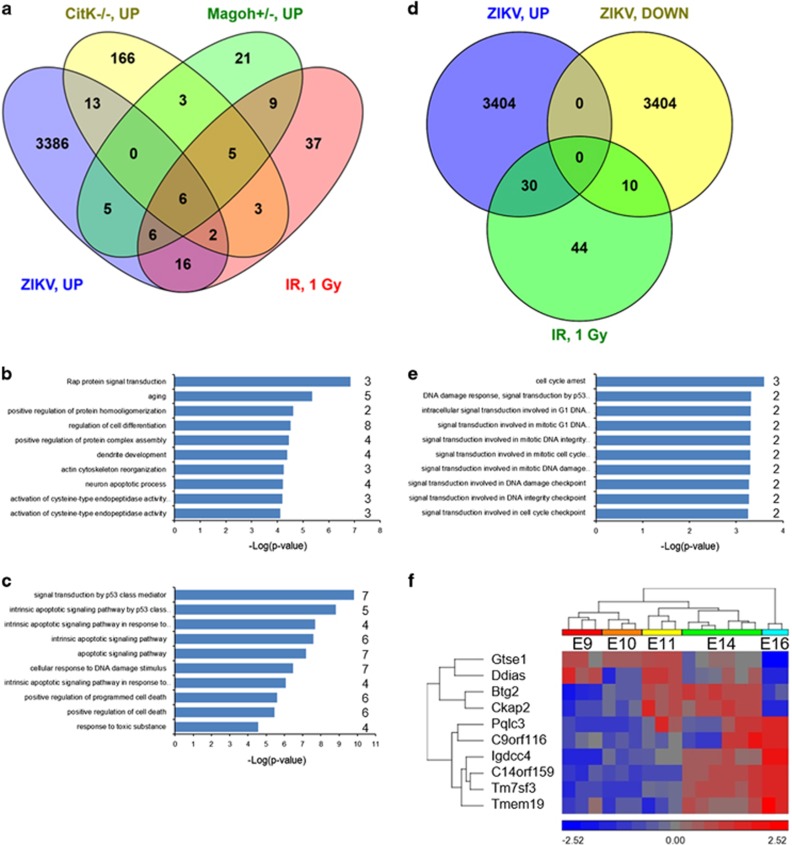
(**a**) Overlap between genes induced in ZIKV-infected hNPCs and different mouse models of microcephaly. Please note that compared to what was published in the study of Di Cunto and co-workers^4^ there is one additional overlapping gene between *Magoh*^+/−^ mice and ZIKV infection, namely *PVT1* (*ENSMUSG00000072566* in mice). (**b**) The 16 genes that are uniquely induced by ZIKV infection and radiation exposure are *APAF1*, *BAIAP2*, *BBC3*, *BLOC1S2*, *ERCC5*, *FAS*, *FOXO3*, *LPIN1*, *NR1D1*, *PLK2*, *PPM1D*, *RAP2A*, *RAP2B*, *STOX2*, *USP32,* and *ZBTB7B*. The bar graph shows top 10 biological processes for which these genes are enriched according to the ToppFun software tool. Numbers behind bars indicate the number of genes from the input list that are involved in these processes. (**c**) Top 10 biological processes enriched among 14 genes that are commonly upregulated between ZIKV-infected hNPCs, genetic microcephaly models, and prenatally irradiated brains. (**d**) Overlap between differentially expressed genes in ZIKV-infected hNPCs and our 84-gene signature. (**e**) Top 10 biological processes enriched among 10 genes that are downregulated in ZIKV-infected hNPCs and upregulated in prenatally irradiated brains. (**f**) Gene expression profiles during mouse embryonic brain development for the 10 genes from (**e**). The data from (**f**) are available at ArrayExpress (E-MTAB-2622). *P*-values represent nominal *P*-values. Only processes with a FDR <0.05 (Benjamini–Hochberg multiple testing correction) were considered significant
